# Combining metformin and nelfinavir exhibits synergistic effects against the growth of human cervical cancer cells and xenograft in nude mice

**DOI:** 10.1038/srep43373

**Published:** 2017-03-02

**Authors:** Chenglai Xia, Ruihong Chen, Jinman Chen, Qianqian Qi, Yanbin Pan, Lanying Du, Guohong Xiao, Shibo Jiang

**Affiliations:** 1 Department of Pharmacy, The Third Affiliated Hospital of Guangzhou Medical University, Guangzhou, 510150, China; 2 Lindsley F. Kimball Research Institute, New York Blood Center, New York, NY 10065, USA; 3 Aris Pharmaceuticals Inc., Bristol, PA19007, USA; 4 Guangdong Provincial Key Laboratory of Reproductive Medicine, Guangzhou, 510150, China; 5 Laboratory of Medical Molecular Virology of Ministries of Education and Health, College of Basic Medical Science, Fudan University, Shanghai, 200032, China

## Abstract

Human cervical cancer is the fourth most common carcinoma in women worldwide. However, the emergence of drug resistance calls for continuously developing new anticancer drugs and combination chemotherapy regimens. The present study aimed to investigate the anti-cervical cancer effects of metformin, a first-line therapeutic drug for type 2 diabetes mellitus, and nelfinavir, an HIV protease inhibitor, when used alone or in combination. We found that both metformin and nelfinavir, when used alone, were moderately effective in inhibiting proliferation, inducing apoptosis and suppressing migration and invasion of human cervical cell lines HeLa, SiHa and CaSki. When used in combination, these two drugs acted synergistically to inhibit the growth of human cervical cancer cells *in vitro* and cervical cancer cell xenograft *in vivo* in nude mice, and suppress cervical cancer cell migration and invasion. The protein expression of phosphoinositide 3-kinase catalytic subunit PI3K(p110α), which can promote tumor growth, was remarkably downregulated, while the tumor suppressor proteins p53 and p21 were substantially upregulated following the combinational treatment *in vitro* and *in vivo*. These results suggest that clinical use of metformin and nelfinavir in combination is expected to have synergistic antitumor efficacy and significant potential for the treatment of human cervical cancer.

According to global data from 2015, cervical cancer is the fourth most common carcinoma among women in developing countries, with more than 130,000 new cases and 50,000 deaths in China alone^1^,^2^,^3^. Surgery is considered the first option for patients with early-stage cervical cancer, but it has limited efficacy for pregnant patients^4^. Several chemical drugs, such as cisplatin, are the first-line treatment used in chemotherapy for cervical cancer. However, some serious side effects, such as bone marrow inhibition, occur during platinum-based chemotherapy^5^,^6^,^7^. Moreover, drug resistance to chemotherapeutics often develops in cervical cancer cells, resulting in tumor recurrence and further progression. Therefore, new chemotherapeutic agents to fight cervical cancer are needed.

HIV protease inhibitors (HIV-PIs) inhibit HIV infection by targeting the viral aspartyl protease. Interestingly, a prospective pilot study showed that regression of Kaposi’s sarcoma (KS) in HIV/AIDS patients occurred during therapy with HIV-PIs^8^. The PI3K/Akt pathway, which is upregulated in HIV-related malignancies associated with Kaposi sarcoma herpes virus (KSHV)^9^, may therefore be suppressed by HIV-PIs^10^. Our previous studies have demonstrated that nelfinavir, an HIV-PI, inhibited the growth of cervical cancer cell lines by promoting apoptosis and arresting the cell cycle at G1 phase^11^, suggesting that nelfinavir may be repositioned as a new therapeutic to treat cervical cancer.

Metformin, a first-line therapeutic drug for type 2 diabetes mellitus, has recently been identified as a potential and attractive anticancer drug^12^. At low doses, metformin effectively inhibited cellular transformation and killed cancer stem cells in breast cancer. Metformin in combination with doxorubicin, a chemotherapeutic agent, could kill both cancer stem cells and cancer cells^13^. It acts as an anti-cancer agent for inhibiting growth of endometrial, ovarian, and cervical cancer cells through mechanism by directly activating AMP-dependent protein kinase (AMPK) and subsequent suppressing mammalian targets of rapamycin (mTOR), thus, inhibiting global protein synthesis and proliferation in target cells^14^,^15^. Although metformin is also effective in inhibiting the growth of cervical cancer cell lines C33A and Me180 through the LKB1-AMPK-mTOR signaling pathway, it is less effective against CaSki and HeLa cells. Knockdown of LKB1 in cervical cells resulted in decreased sensitivity to metformin, suggesting that metformin can be used as a potential therapeutic to treat cervical cancers, particularly those with normal LKB1 expression^16^.

Growing evidence suggests that the combination of cancer chemo-preventive and- therapeutic agents may produce synergistic effects with anticancer efficacy, resulting in the decreased concentration of each drug used in the combination and reduced side effects when compared with the drugs used in mono-therapy. It is widely believed that high-risk types of human papillomavirus (HPV), such as HPV-16 and HPV-18, are the main causative agents of human cervical cancer. In the present study, we used an HPV-18-infected human cervical cell line (HeLa) and two HPV-16-infected human cervical cell lines (SiHa and CaSki) to investigate the potential synergistic anticancer effect of metformin and nelfinavir, as a combinational therapy, in addition to the mechanisms underlying such synergy.

## Results

### Combining nelfinavir and metformin exhibits synergistic effect against the growth of cervical cancer cells

We tested the inhibitory activity of nelfinavir, metformin and the nelfinavir/metformin combination on proliferation of human cervical cell lines CaSki, SiHa, and HeLa, using XTT assay. As shown in Table 1, nelfinavir alone inhibited the growth of CaSki, SiHa, and HeLa cells with IC50s of 11.44, 16.49, and 26.47 μM, respectively, while metformin alone suppressed CaSki, SiHa, and HeLa cell growth with IC50s of 39.23, 68.38, and 223.23 mM, respectively. However, the combination of metformin and nelfinavir exhibited synergistic effect with combination index (CI) of 0.38, 0.61, and 0.21 for inhibiting 50% growth of CaSki, SiHa, and HeLa cells, respectively. In combination, IC50s of nelfinavir for inhibiting CaSki, SiHa, and HeLa cell growth were 2.02, 5.11, and 3.31 μM, respectively, with a dose reduction of approximately 3- to 9-fold. In combination, the IC50s of metformin for inhibiting CaSki, SiHa, and HeLa cell growth were 8.08, 20.44, and 12.57 mM, respectively, with a dose reduction of 3- to 18-fold. These results suggest that combinational use of these two drugs may have synergistic effect against cervical cancer cell growth.

**Figure 1:**
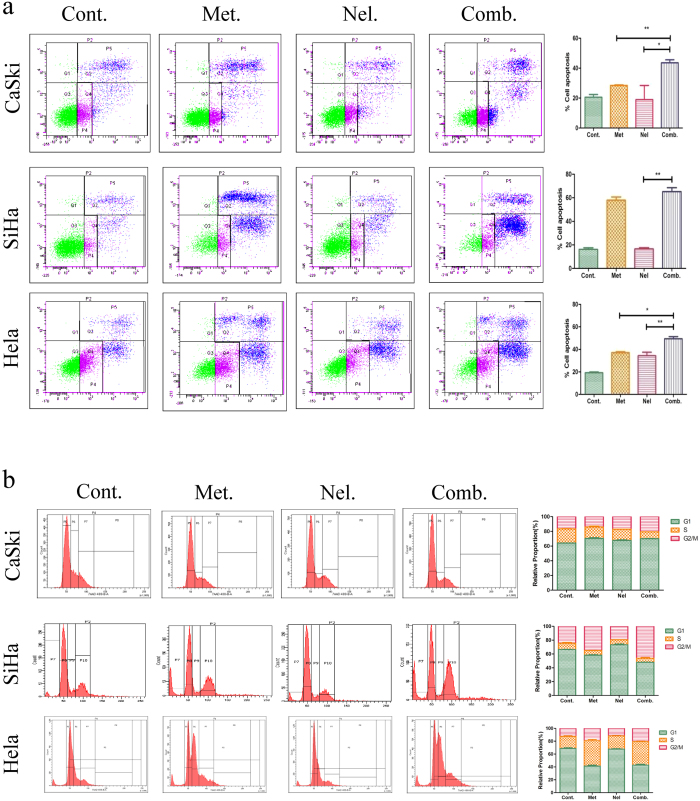
Inducing apoptosis of cervical cancer cell lines by metformin and nelfinavir, alone or in combination. (**a**) Analysis of apoptotic cells by flow cytometry. SiHa, HeLa and CaSki cells were stained with Annexin-V-FITC and PI following treatment with or without 10 mM metformin and 4 μM nelfinavir, alone or in combination, for 72 h. Apoptosis was determined by flow cytometry. (**b**) Analysis of cell cycle arrest. SiHa, HeLa and CaSki cells were treated with metformin and nelfinavir, alone or in combination, at the indicated concentrations for 72 h. The cells were stained with 7-AAD and analyzed by flow cytometry. The test for synergy: *p < 0.05; **p < 0.01, compared to untreated control or individual drug (Cont, control; Met, metformin; Nel, nelfinavir; Comb, the combination of metformin and nelfinavir).

Apoptosis is an active process of cell death characterized by cell shrinkage, chromatin aggregation with genomic fragmentation, and nuclear pyknosis. Promoting cancer cell apoptosis is a key characteristic of chemotherapy drugs^17^,^18^. To test the combined effect of metformin and nelfinavir on apoptosis of human cervical cells, we used Annexin V/PI staining to measure apoptosis following metformin and nelfinavir treatment, alone or in combination. The ratio of apoptotic cells in CaSki cells treated with PBS as control was 20.65 ± 1.76%, while the ratio of apoptotic cells in CaSki cells treated with metformin or nelfinavir alone was 28.45 ± 0.35%, or 19.00 ± 9.48%, respectively. This suggests that metformin alone is able to induce some apoptosis, whereas nelfinavir alone does not. However, the ratio of apoptotic cells in the CaSki cells treated with the metformin/nelfinavir combination was 43.60 ± 1.98%. This ratio is significantly higher than that of CaSki cells treated with either metformin or nelfinavir alone, indicating that the combination of metformin and nelfinavir has synergistic effect on the induction of apoptosis in human cervical cells. A similar effect was also observed when SiHa and HeLa cell lines were tested (Fig. 1a).

Like other tumors, the occurrence of cervical cancer is induced by dysregulated cell cycle, which results in the abnormal proliferation and differentiation of cells. Chemotherapy drugs often cause cancer cell cycle arrest^19^,^20^. We used 7-AAD staining to investigate the effect of the metformin and nelfinavir combination on the cell cycle of cervical cancer cells. As shown in Fig. 1b, no significant difference in cell cycle arrest is seen in the CaSki cell line, but when metformin and nelfinavir are combined, we see a clearly increased cell cycle G2/M phase arrest compared to each drug alone in the SiHa and HeLa cell lines, suggesting that the combination of metformin and nelfinavir, when compared to either drug alone, has a stronger anticancer effect by causing cancer cell cycle arrest in some cervical cancer cells.

### Combining metformin with nelfinavir shows strong synergistic inhibition of human cervical cancer cell migration and invasion

**Figure 2:**
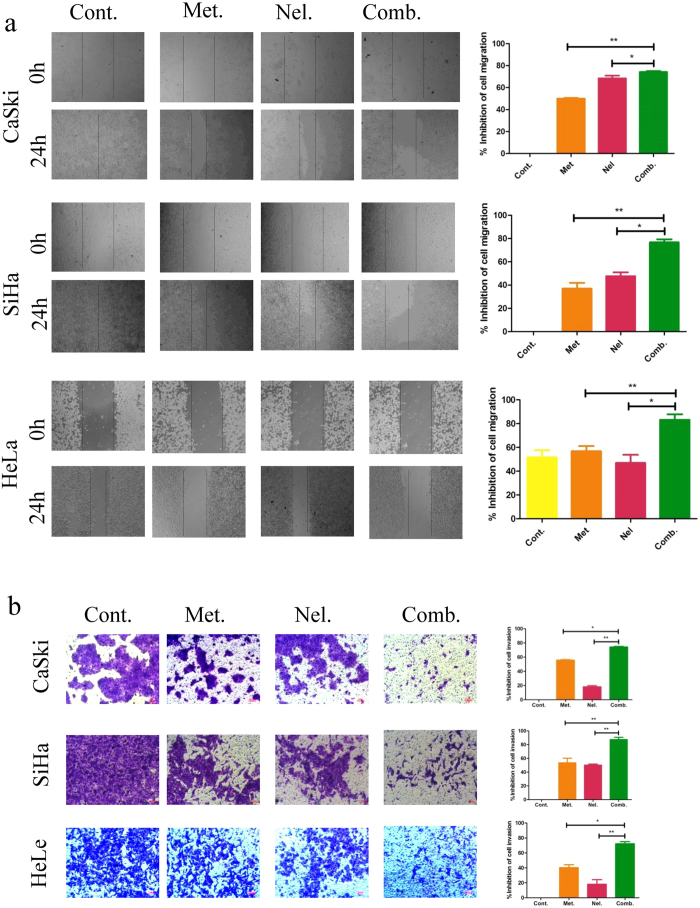
Inhibition of cell migration and invasion by metformin and nelfinavir, alone or in combination. (**a**) A wound-healing assay was performed to measure cell migration. SiHa, HeLa and CaSki cells were seeded in 24-well plates in the absence or presence of metformin and nelfinavir, alone or in combination, and the wound was generated by scratching the surface of the plates with a 10 μl pipette tip. Cells were then washed with PBS and incubated with metformin and nelfinavir, alone or in combination, at the indicated concentrations for 24 h. (**b**) For invasion assay, the invasive capacity of SiHa, HeLa and CaSki cells in the absence or presence of metformin and nelfinavir, alone or in combination, after 48hwas assessed using the Matrigel invasion chamber. Each sample was tested in triplicate and data are shown as means ± SEM. The test for synergy: *p < 0.05; **p < 0.01, compared to untreated control or individual drug (Cont, control; Met, metformin; Nel, nelfinavir; Comb, the combination of metformin and nelfinavir).

Metastasis is a key process in cancer progression, and cancer cells in a primary tumor acquire invasive properties and disseminate to other sites in the body to initiate secondary lesions. Most human malignant tumor cells, particularly cervical cancer, or other epithelial malignant tumor cells, collectively proliferate and invade into the stroma, forming groups, nests and tubular architecture. Cell migration is a key characteristic of cancer cells. Many chemotherapy drugs inhibit cancer cell migration^21^,^22^. To analyze the effects of the metformin and nelfinavir combination on human cervical cancer cell migration and invasion, we used wound-healing assays and a precoated Matri gel invasion chamber to measure the effects of the metformin and nelfinavir combination on human cervical cancer cells. As shown in Fig. 2a, the percent inhibition of cell migration in the CaSki cell line treated with metformin or nelfinavir alone was 49.99 ± 0.54% or 68.38 ± 2.6%, respectively, while the percent inhibition of cell migration in the CaSki cells treated with a combination of metformin and nelfinavir was 74.23 ± 0.8%, which is significantly higher than metformin alone (P < 0.01) or nelfinavir alone (P < 0.05). Similar results were obtained with HeLa and SiHa cell lines.

As shown in Fig. 2b, the percent inhibition of cell invasion in the CaSki cell line treated with metformin or nelfinavir alone was 55.8 ± 0.55% or 18.1 ± 1.6%, respectively, while percent inhibition of cell invasion in the CaSki cells treated with a combination of metformin and nelfinavir was 74.2 ± 0.87%, which is significantly higher than metformin alone (P < 0.01) or nelfinavir alone (P < 0.05). Similar results were obtained with SiHa and HeLa cell lines. These results suggest that the combination of metformin and nelfinavir has synergistic effect against the migration and invasion of cervical cancer cells and possibly also against metastasis of cervical carcinoma.

### Combining metformin and nelfinavir leads to synergistic effect on upregulating ROS and p53 and p21expression and downregulating PI3K(p110α) expression in cervical cancer cells

**Figure 3:**
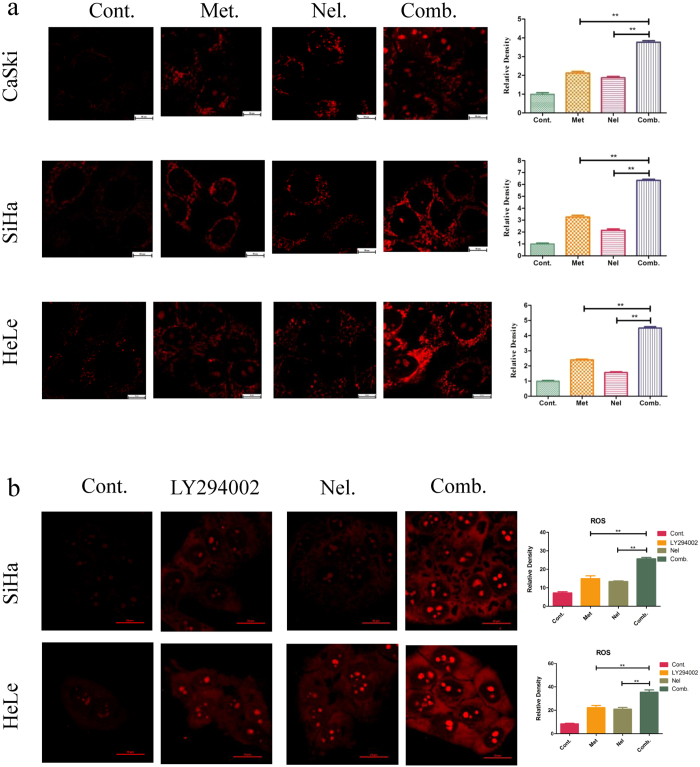
Inducing ROS production in SiHa, HeLa and CaSki cells by metformin (**a**) or LY294002 (**b**) and nelfinavir, alone or in combination a. SiHa, HeLa and CaSki cells were treated with 10 mM metformin or 4 μM nelfinavir, alone or in combination, for the indicated times. MitoSOX™ Red mitochondrial superoxide indicator. Each sample was tested in triplicate and data are shown as means ± SEM. Values are expressed as the fold change of untreated control at the indicated time point. The test for synergy: *p < 0.05; **p < 0.01, compared to untreated control or individual drug (Cont, control; Met, metformin; Nel, Nelfinavir; Comb, the combination of metformin and nelfinavir).

ROS are produced by various biochemical and physiological oxidative processes in the body which are associated with numerous physiological and pathophysiological processes. Recent findings have indicated that ROS play a crucial role in the survival of cancer cells^23^,^24^. Some classical anticancer chemical drugs, such as paclitaxel and adriamycin, can induce cancer cell apoptosis via stimulating the production of mitochondrial ROS^25^,^26^. To investigate the effect of the metformin and nelfinavir combination on ROS production in cancer cells, we measured the mitochondrial ROS levels in CaSki cells using laser confocal microscopy. We found that the metformin/nelfinavir combination could significantly induce mitochondrial ROS production, compared to either drug alone. We also observed the same phenomenon in SiHa and HeLa cell lines (Fig. 3a). LY294002, a phosphoinositide 3-kinase inhibitors, could direct inhibit the PI3K/Akt signaling pathway, like metformin^27^,^28^. Here we used LY294002 as a control for metformin to investigate the effect of the LY294002/nelfinavir combination on ROS production in cervical cancer cells. We found that the LY294002/nelfinavir combination could significantly induce mitochondrial ROS production, compared to either drug alone (Fig. 3b). These findings suggest that the metformin and nelfinavir combination may induce mitochondrial ROS production in cervical cancers.

**Figure 4:**
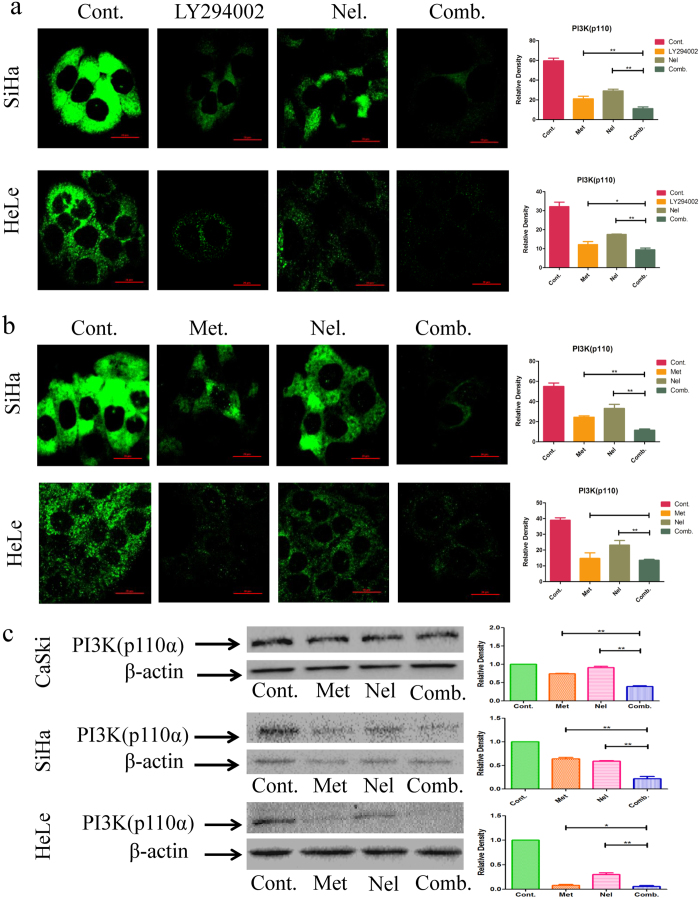
The effects of metformin (LY294002 as a control) and nelfinavir, alone or in combination, on cancer-related proteins PI3K(p110α). SiHa, HeLa and CaSki cells were treated with 10 mM metformin or 50 μM LY294002 or 4 μM nelfinavir, alone or in combination, for the indicated times. Antibodies against PI3K were used. (**a,b**) The expression of PI3K(p110α) was measured by confocal microscope. (**c**) The expression of PI3K(p110α) was measured by Western blot. Each sample was tested in triplicate and data are shown as means ± SEM. Values are expressed as the fold change of untreated control at the indicated time point. *p < 0.05, **p < 0.01, compared to untreated control or individual drug (Cont, control; Met, metformin; Nel, Nelfinavir; Comb, the combination of metformin and nelfinavir).

**Figure 5:**
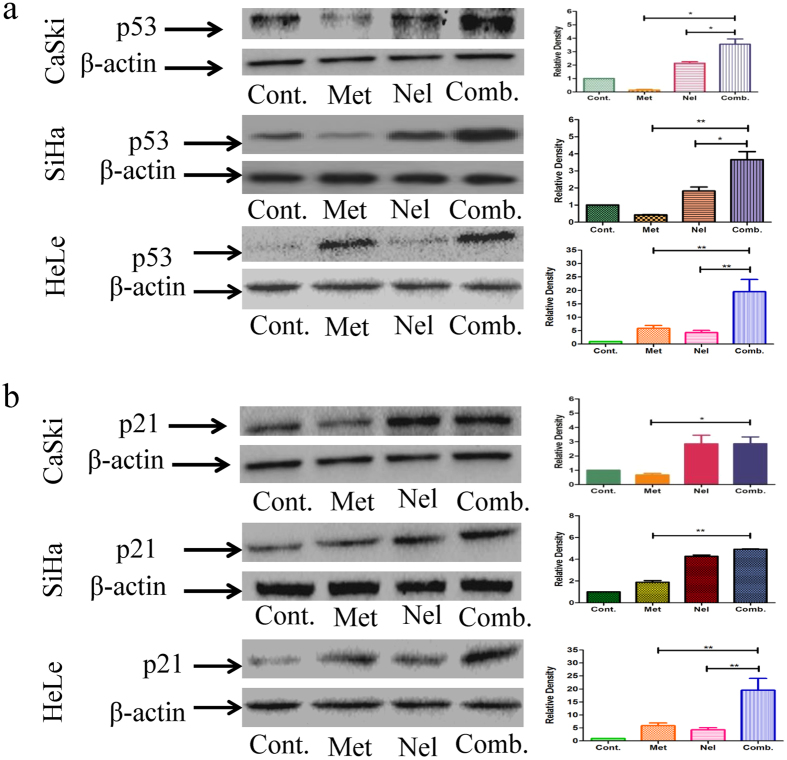
The effects of metformin and nelfinavir, alone or in combination, on cancer-related proteins p53 (**a**) and p21 (**b**). SiHa, HeLa and CaSki cells were treated with 10 mM metformin or 4 μM nelfinavir, alone or in combination, for the indicated times. Antibodies against p53 and p21 were used. Each sample was tested in triplicate and data are shown as means ± SEM. Values are expressed as the fold change of untreated control at the indicated time point. *p < 0.05, **p < 0.01, compared to untreated control or individual drug (Cont, control; Met, metformin; Nel, Nelfinavir; Comb, the combination of metformin and nelfinavir).

Recent studies on molecular profiling have revealed that the phosphatidylinositol 3-kinase (PI3K)/Akt/mTOR pathway mediates multiple cellular functions^29^. Activation of the PI3K/Akt signaling pathway is critical for tumor cell growth and survival in a number of solid tumors^30^. To further investigate the combinatorial effect of metformin and nelfinavir on cancer-related proteins, we measured the expression of PI3K(p110α), p53 and p21in SiHa, CaSki and HeLa cells after treatment with metformin and nelfinavir, alone or in combination. As a control for metformin, we investigated the effect of the LY294002/nelfinavir combination on inhibition of PI3K(p110α) in cancer cells by confocal microscopy. We found that the LY294002/nelfinavir combination could significantly inhibit PI3K(p110α) expression in SiHa and Hela cells, compared to either drug alone (Fig. 4a). We also found that P13K(p110α) protein expression was significantly reduced in these cervical cancer cells treated with metformin or nelfinavir alone, while its expression in SiHa, CaSki and Hela cells treated with the combination of metformin and nelfinavir was more significantly inhibited (Fig. 4b,c). Interestingly, metformin treatment resulted in the decreased expression of p53 in SiHa and CaSki, but increased p53 expression in HeLa cells. However, the combined metformin/nelfinavir treatment led to a significant increase of p53 expression in SiHa, CaSki and HeLa cell lines (Fig. 5a). A similar phenomenon was also observed for the expression of p21protein in these cervical cancer cells (Fig. 5b). These results suggest that the metformin and nelfinavir combination has synergistic effect, inhibiting PI3K expression and enhancing p53/p21protein expression.

### Combining metformin and nelfinavir has strong synergistic effect against the growth of cervical cancer cell xenograft in nude mice

**Figure 6:**
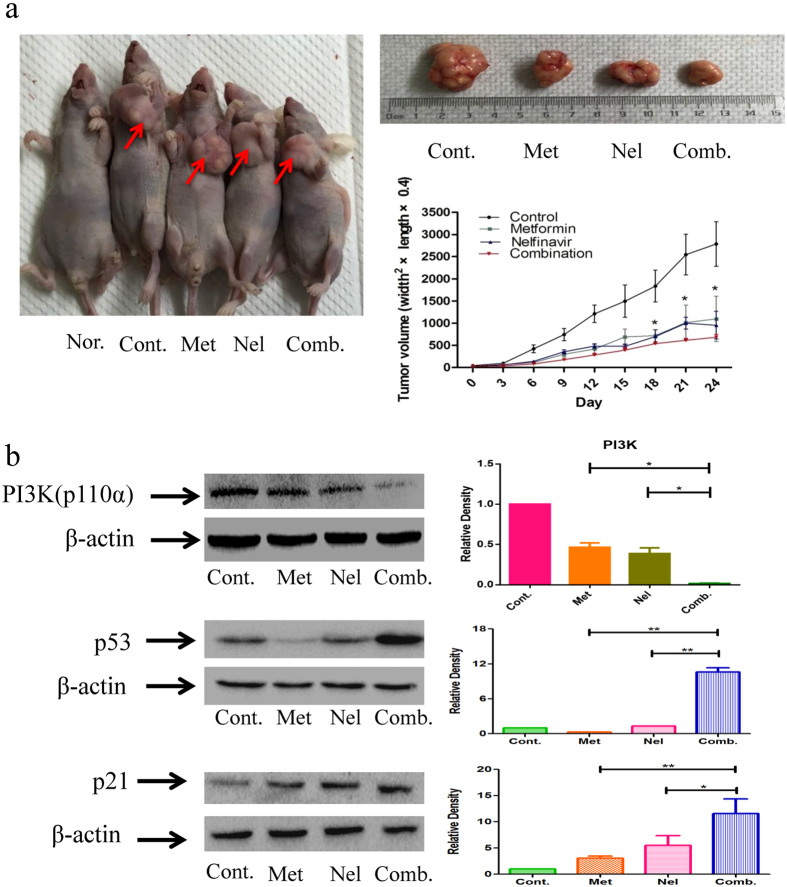
Inhibition of tumor growth in mouse model by metformin and nelfinavir, alone or in combination. (**a**) Inhibition of cervical tumor growth. SiHa cells (1 × 10^6^) suspended in PBS were injected subcutaneously into the left flanks of female nude mice (BALB/c). The mice with tumors (0.3–0.4 cm wide and 0.3–0.4 cm long) were randomly assigned to each treatment and were injected i.p. with vehicle (5 μL/g body weight), metformin (100 mg/kg), nelfinavir (0. 4 mg/kg), or metformin (100 mg/kg) plus nelfinavir (0. 4 mg/kg) three times per week for 24 days (n = 5 per group). Tumor size (length and width) was measured before each i.p. injection, and tumor volume was calculated using the following formula: width^2^ × length × 0.4. The right panel shows the tumor volume in each group by day, and the middle and left panels show representative images of the tumors. Values are shown as means ± SE, n = 5. (**b**) Protein expression of PI3K, p53 and p21 in tumor tissues. The protein expression of PI3K, p53 and p21 in tumor tissues was analyzed by Western blotting with the indicated antibodies. Values are expressed as the fold change of the vehicle-treated control and are shown as the mean ± SE, n = 5. *p < 0.05, **p < 0.01, ^##^p < 0.01, compared to untreated control or individual drug (Nor, Normal; Mod, model; Met, metformin; Nel, nelfinavir; Comb, the combination of metformin and nelfinavir).

To investigate the combinational effect of metformin and nelfinavir on tumor growth *in vivo*, we established a xenograft model in BALB/c nude female mice injected with SiHa cells and then treated with metformin (100 mg/kg body weight) and nelfinavir (0.4 mg/kg body weight), alone or in combination, for 24 days. As shown in Fig. 6a, the tumor volume in the combination treatment group was significantly smaller than that in the single-drug treatment groups, suggesting that the combination of metformin and nelfinavir significantly suppressed the growth of SiHa cells in BALB/c mice. We also measured PI3K(p110α), p53 and p21 protein expression in xenograft tumor tissues. Compared to the single-drug treatment groups, the expression of PI3K(p110α) significantly decreased, while the expression of p53 and p21remarkably increased in the combination treatment group (Fig. 6b), which is consistent with the *in vitro* assessment. These findings suggest that the combination of metformin and nelfinavir inhibits tumor growth in mice, possibly through inhibition of PI3K(p110α) expression and increase of p53/p21expression in cervical cancer cells.

## Discussion

Recent epidemiological studies have demonstrated that diabetic patients treated with metformin have reduced cancer incidence and mortality^31^,^32^. Growing evidence gained from *in vitro* and *in vivo* studies has indicated the direct effect of metformin on many types of cancer cells, and its IC_50_ value is approximately 50 mM^33^. Moreover, metformin can inhibit PI3K/Akt/mTOR signal pathway expression and has been shown to have chemopreventive effects against cervical cancer and is currently being explored as a therapeutic option with both indirect (i.e., insulin-dependent) and direct (i.e., insulin-independent) mechanism of action against a variety of cancer types^34^.

Several HIV protease inhibitors were reported to have direct antitumor activities against lung cancer^35^, breast cancer^36^, glioblastoma^37^, melanoma^38^, multiple myeloma^39^ and leukemia^40^. Our previous studies have shown that nelfinavir, a HIV protease inhibitor, inhibits the growth of cervical cancer cell lines (SiHa, HeLa, and CaSki) by promoting apoptosis and arresting the cell cycle at G1 phase^11^.

It is well established that combinatorial therapies consisting of anticancer drugs with different mechanisms of action result in synergistic effect that is generally more effective than monotherapy^41^,^42^. Since metformin and nelfinavir inhibit the growth of cervical cancer cells by different mechanisms of action, we hypothesized that combining metformin and nelfinavir could have synergistic effects against human cervical cancer cell growth. Indeed, our results demonstrated that the metformin/nelfinavir combination exhibited significantly higher inhibition than either metformin or nelfinavir alone on *in vitro* growth of human cervical cancer cell lines CaSki, SiHa, and HeLa, as well as *in vivo* growth of SiHa xenograft tumor in nude mice, resulting in a significant dose reduction of each drug tested in the combination.

We then studied the underlying mechanisms by which the metformin/nelfinavir combination inhibits cancer cell growth. Apoptosis is characterized by a series of biochemical and morphological changes. One of the most significant events in apoptosis is mitochondrial dysfunction and ROS overproduction^43^. Our previous studies have shown that nelfinavir induced apoptosis of cervical cancer cells through the enhancement of mitochondrial ROS production^11^. To explore the detailed molecular mechanism by which the metformin/nelfinavir combination inhibits human cervical cancer, we used confocal microscopy and Western blot analyses to determine if mitochondrial ROS levels were altered following treatment with metformin alone or in combination with nelfinavir. Results showed that the combination treatment induced a higher level of mitochondrial ROS production in cervical cancer cells than the treatment with metformin alone or nelfinavir alone. We found that the LY294002/nelfinavir combination could significantly induce ROS production, compared to either drug alone. These findings suggest that the metformin/nelfinavir combination has synergistic effect through enhancing mitochondrial ROS production in cervical cancer cells, thereby inducing an elevated level of cervical cancer cell apoptosis.

In the last two years, it has been reported that the PI3K/Akt/mTOR signaling pathway plays a central role in growth, metabolism, survival, and motility of cancer cells, making it an attractive target for antitumor drug development^44^. Inhibition of signaling along this pathway can lead to decreased cell proliferation and increased cell death^45^. In endometrial cancer cells, metformin induced G1 arrest and caused apoptosis by suppressing mTOR signaling^46^. The tumor suppressor protein p53, encoded by the TP53 gene, executes its function by inducing cell cycle arrest and apoptosis in response to DNA damage. Cell cycle arrest driven by p53 requires the transcription of p21, which is a cyclin-dependent kinase inhibitor. In general, DNA damage or stress increases the levels of p53 protein, in turn inducing p21 transcription and leading to cell cycle arrest at G1 or G2^47^.

To examine the indirect chemopreventive effects of metformin that might be enhanced by nelfinavir, we analyzed key proteins of the PI3K/Akt/mTOR axis in both human cervical cell line and mouse cervical tumor tissue. PI3Ks are composed of a catalytic subunit (p110) and a regulatory subunit. Various isoforms of the catalytic subunit (p110α, p110β, p110γ, and p110δ) have been isolated and reported^27^. LY294002 is a phosphoinositide 3-kinase inhibitor, which inhibits the PI3K/Akt signaling pathway^27^. Here, we found that the LY294002/nelfinavir combination significantly inhibited PI3K(p110α) expression, compared to either drug alone.

In general, combining two active molecules having the same target in a signal pathway may result in antagonistic effect, while combination of two active molecules having different targets may lead to synergistic effect^48^. It has been demonstrated that nelfinavir downregulates the P13K/Akt/mTOR signal pathway by targeting Akt^49^, while metformin downregulates the P13K/Akt/mTOR signal pathway by targeting mTOR^50^. Therefore, combining nelfinavir and meteform resulted in synergistic effect on downregulation of the P13K/Akt/mTOR signal pathway and indirect suppression of P13K protein expression.

Moreover, the metformin/nelfinavir combination is significantly more effective in increasing the expression of p53 and p21 in cervical cancer cell lines and tumor tissues than either metformin alone or nelfinavir alone. It was recently reported that treatment of cancer cells with metformin resulted in increased p53 protein expression and then enhanced transcription of its downstream target genes, Bax and p21. The metformin-induced p53 up-regulation is through the AMPK-mTOR signaling pathway^51^. Nelfinavir can also upregulate p53 and p21 expression, but via a different mechanism, i.e., inhibition of E6-mediated proteasomal degradation of p53 in HPV-transformed cervical carcinoma cells^52^. Because metformin and nelfinavir use different mechanisms to upregulate the expression of p53 and its downstream product, p21, their combination thus exhibits synergistic effect on p53 and p21 expression. These results suggest that because of the above synergistic mechanisms, the combination of metformin and nelfinavir exhibits synergistic effect on the inhibition of cervical cancer cell DNA replication and induction of DNA damage to promote apoptosis and cell cycle arrest.

In summary, the combination of metformin and nelfinavir has shown significant synergistic effect against cervical cancer cell growth, resulting in meaningful dose reduction for each drug in the combination. Because both metformin and nelfinavir are FDA-approved drugs, their clinical use is supported by significant safety profiles. Therefore, repositioning them as antineoplastic agents (alone or in combination) and developing them as a safe and effective combination regimen for introduction to the market place are expected to occur at a much faster pace than the development of totally new combinational compounds. Moreover, this approach can be applied to the development of other combinational anticancer therapies against not only cervical cancer, but also other tumors for which no effective antiviral agents are currently available.

## Materials and Methods

### Cell viability assays

As previously described^53^, cell viability was measured using XTT assays. Briefly, cervical cancer HeLa, SiHa and CaSki cells were seeded in 96-well plates (10^4^ cells per well). Cells were cultured in the presence or absence of metformin and nelfinavir at graded concentration for 72 h. A 2,3-bis [2-methoxy-4-nitro-5-sulfophenyl]-5-[(phenylamino) carbonyl]-2H-tetrazolium hydroxide (XTT) working solution was added to the cultured cells and incubated for an additional 4 h. Absorbance intensity was measured by a ELISA microplate reader (MultiscanEX, Labsystems, Finland) at 450 nm. Cell viability is presented as an optical density (OD) ratio (treated to untreated cells).

### Cell apoptosis assay

Cervical cancer HeLa, SiHa and CaSki cells were seeded at a density of 2 × 10^5^ cells/well in a 6-well culture plate for 3 days. Cells were washed twice with cold PBS and then resuspended in 1X binding buffer. Then, 100 μl of the cell suspension (1 × 10^5^ cells) were transferred to a 5 ml culture tube and mixed with 5 μl of FITC-Annexin V and 5 μl PI in the presence or absence of metformin and nelfinavir. The mixture was gently vortexed and incubated for 15 min at RT (25 °C) in the dark. Then, 400 μl of 1X binding buffer were added to each tube. The cells were analyzed by flow cytometry within 1 h. The green fluorescence of Annexin V-FITC was measured at 530 nm, and the red fluorescence of PI was measured at 585 nm. The results were analyzed with FlowJo software.

### Cell migration and invasion assay

Human cervical cancer SiHa, CaSki and HeLa cells were cultured in 24-well plates and placed into an incubator (37 °C, 5% CO_2_) until they reached 90% confluence. Cell migration was measured using a wound healing assay. In brief, scratch wounds of the same width on each cell monolayer were created with a sterile 10 μl tip. The detached cells were removed by washing with phosphate-buffered saline (PBS). Cells were then treated with metformin and nelfinavir, alone or in combination, to block cellular migration. Photos were taken at 24 h, and the distance traveled by the cells indicated the closure of the wounds. Cell invasion was measured using a 24-well, 8-μm pore size Transwell chamber assay (Corning Inc., Corning, NY) according to the manufacturer’s protocol. In brief, filters were precoated on the upper side with Matrigel (1 mg/mL; BD Biosciences, San Jose, CA). The lower chamber was filled with culture media containing 10% FBS. Cells (2 × 10^5^) were seeded in the upper chambers for appropriate hours at 37 °C. After incubation, cells invading the bottom surface of the filter were fixed in methanol and stained with 0.1% crystal violet. The invading cell number was quantified by counting at least six random fields (200× magnification). Each sample was tested in triplicate and the *in vitro* experiment was repeated at least twice.

### Cell cycle arrest assay

As described in a previous study^54^, SiHa, CaSki and HeLa cells were harvested in PBS and fixed in ice-cold 70% ethanol, which was added with a Pasteur pipette with vortexing. Then, the cells were centrifuged at approximately 2,000 rpm for 5 min and washed twice in PBS. Finally, the cells were stained with 7-AAD staining solution (Invitrogen) and analyzed by flow cytometry (collecting 25,000 events per sample).

### Mitochondrial reactive oxygen species (ROS) assays

Mitochondrial ROS levels in the cervical cancer cell lines were measured via MitoSOX^TM^ Red assays based on the manufacturer’s instructions. Briefly, SiHa, CaSki and HeLa cells were incubated in HBSS with MitoSOX^TM^ Red at a final concentration of 5 μM for 15 min at 37 °C and then washed with HBSS. Fluorescence intensity was imaged using confocal microscopy (Zeiss 510 Meta).

### Cell immunofluorescence staining

Fix and permeabilize Human cervical cancer SiHa, CaSki and HeLa cells on coverslips in the 12-well plate. After washing with TBST, the cells was incubated with PI3K (p110α) antibody (Cell Signaling Technology, Danvers, MA) and FITC-Labeled secondary antibodies (Cell Signaling Technology, Danvers, MA). Images were visualized with confocal microscopy in response to test conditions.

### Western blot

Following treatments, cells were washed twice with ice-cold PBS and harvested in sample buffer. Soluble extracts were prepared by centrifugation at 12,000 g for 30 min at 4 °C. Protein concentration was determined using a BCA kit (Pierce Chemical Co., Rockford, IL). After separation by SDS-PAGE, proteins were transferred to nitrocellulose membranes (Bio-Rad, Hercules, CA). Membranes were incubated in TBST containing 5% nonfat milk for 1 h at room temperature. The blots were then reacted with primary antibodies overnight at 4 °C. Antibodies used for Western blotting analysis included PI3K(p110α), p53 and β-actin antibody (Cell Signaling Technology, Danvers, MA). After washing with TBST, the membrane was incubated with the corresponding horseradish peroxidase-conjugated secondary antibodies (Cell Signaling Technology, Danvers, MA). The signals were visualized with ECL (Pierce Chemical Co.) and then exposed with ChemiDoc MP (Hercules, CA).

### *In vivo* tumor experiments

Female nude mice (BALB/c, 4–5 weeks old) were obtained from the Animal Center of Guangdong Province [License No. SCXK (Yue) 2006–0015)]. All animal protocols were reviewed and approved by the Ethics Committee of the Third Affiliated Hospital of Guangzhou Medical University. Single-tumor cell suspensions (1 × 10^6^) were injected subcutaneously into the left flank of each mouse to obtain cervical cancer xenografts. The mice were divided into four groups (5 mice/group) 5 days after cell implantation. Mice in the experimental groups were intraperitoneally injected with metformin at a dose of 100 mg/kg body weight per day, nelfinavir (100 mg/kg body weight per day) or a combination of metformin and nelfinavir. Mice in the control group received an equal volume of normal saline. Tumor volume was evaluated every two days by two cross-sectional measurements, and tumor size was calculated as follows: tumor volume = width^2^ × length × 0.4. Mice were sacrificed after 24 days, and the tumors were weighed and then fixed in 10% formalin for the following experiments. Mouse care and use were performed in accordance with local ethical guidelines.

### Statistical analyses

Data are expressed as means ± SD. The data were assessed with independent *t*-test or ANOVAs, as appropriate. P < 0.05 indicated significance. All statistical analyses were performed with SPSS 19.0 software.

## Additional Information

**How to cite this article:** Xia, C. *et al*. Combining metformin and nelfinavir exhibits synergistic effects against the growth of human cervical cancer cells and xenograft in nude mice. *Sci. Rep.* **7**, 43373; doi: 10.1038/srep43373 (2017).

**Publisher's note:** Springer Nature remains neutral with regard to jurisdictional claims in published maps and institutional affiliations.

## Figures and Tables

**Table 1:** Combination index and dose reduction value for inhibition of cervical carcinoma cells.

Inhibition (%)	CI*	Nelfinavir				Metformin			
		Con.(μM)		Dose reduction	P	Con. (mM)		Dose reduction	P
		Alone Mix				Alone Mix			
CaSki cells									
50	0.38	11.44	2.02	5.65	0.007	39.23	8.09	4.85	0.003
70	0.38	14.84	2.52	5.88	0.009	48.89	10.09	4.85	0.045
90	0.37	22.46	3.58	6.27	0.015	69.42	14.33	4.84	0.002
95	0.35	46.90	6.68	7.02	0.031	129.39	26.73	4.84	0.026
Siha cells									
50	0.61	16.49	5.11	3.23	0.005	68.38	20.44	3.35	0.013
70	0.56	23.38	6.00	3.90	0.002	78.81	24.00	3.28	0.016
90	0.50	40.81	7.75	5.27	0.001	98.80	30.99	3.19	0.001
95	0.44	109.72	12.20	9.00	0.000	147.61	48.78	3.03	0.007
HeLa cells									
50	0.21	26.47	3.31	8.00	0.022	223.23	12.57	17.76	0.007
70	0.21	31.75	3.96	8.01	0.024	262.74	14.74	17.83	0.010
90	0.21	42.44	5.29	8.03	0.027	340.64	19.00	17.93	0.015
95	0.20	71.07	8. 82	8.06	0.034	540.27	29.82	18.12	0.024

SiHa, HeLa and CaSki cells were treated with 10 mM metformin or 4 μM nelfinavir, alone or in combination, for the indicated times. *CI, combination index. A CI of >1, 1, and <1 indicates antagonism, additive effect, and synergism, respectively. The strength of synergism: very strong synergism (CI: <0.1); strong synergism (CI: 0.1–0.3); synergism (CI: 0.3–0.7); moderate synergism (CI: 0.7–0.85); and slight synergism (CI: 0.85–0.90). “Dose reduction (fold)” = the IC_50_ value of an inhibitor tested alone/the IC_50_ value of the same inhibitor tested in combination with another inhibitor.
